# More of what? Dissociating effects of conceptual and numeric mappings on interpreting colormap data visualizations

**DOI:** 10.1186/s41235-023-00482-1

**Published:** 2023-06-19

**Authors:** Alexis Soto, Melissa A. Schoenlein, Karen B. Schloss

**Affiliations:** 1 Department of Integrative Biology, University of Wisconsin-Madison, 430 Lincoln Drive, Madison, WI 53706 USA; 2 Department of Psychology, University of Wisconsin-Madison, 1202 W. Johnson Street, Madison, WI 53706 USA; 3 Wisconsin Institute for Discovery, University of Wisconsin-Madison, 330 N. Orchard Street, Madison, WI 53715 USA

**Keywords:** Information visualization, Color cognition, Visual reasoning

## Abstract

In visual communication, people glean insights about patterns of data by observing visual representations of datasets. Colormap data visualizations (“colormaps”) show patterns in datasets by mapping variations in color to variations in magnitude. When people interpret colormaps, they have expectations about how colors map to magnitude, and they are better at interpreting visualizations that align with those expectations. For example, they infer that darker colors map to larger quantities (dark-is-more bias) and colors that are higher on vertically oriented legends map to larger quantities (high-is-more bias). In previous studies, the notion of quantity was straightforward because more of the concept represented (conceptual magnitude) corresponded to larger numeric values (numeric magnitude). However, conceptual and numeric magnitude can conflict, such as using rank order to quantify health—smaller numbers correspond to greater health. Under conflicts, are inferred mappings formed based on the numeric level, the conceptual level, or a combination of both? We addressed this question across five experiments, spanning data domains: alien animals, antibiotic discovery, and public health. Across experiments, the high-is-more bias operated at the conceptual level: colormaps were easier to interpret when larger conceptual magnitude was represented higher on the legend, regardless of numeric magnitude. The dark-is-more bias tended to operate at the conceptual level, but numeric magnitude could interfere, or even dominate, if conceptual magnitude was less salient. These results elucidate factors influencing meanings inferred from visual features and emphasize the need to consider data meaning, not just numbers, when designing visualizations aimed to facilitate visual communication.

**Supplementary Information:**

The online version contains supplementary material available at 10.1186/s41235-023-00482-1.

## Significance

Visual communication is fundamental to sharing of information across sectors, spanning academia, politics, business, and general public discourse. People use various kinds of information visualizations to communicate about data, but colormap data visualizations (“colormaps”) are especially useful for showing how patterns of data unfold over space. In colormaps, gradations of color correspond to gradations of quantity over space. Common examples include maps of weather patterns across a city, election outcomes across a country, and disease prevalence across the globe. When people interpret colormaps, they have expectations about how colors will map to quantities, and it is harder to interpret colormaps that violate those expectations. Several biases contribute to inferences about the meanings of colors in colormaps. For example, the dark-is-more bias leads to the inference that darker colors map to larger quantities, and the high-is-more bias leads to the inference that colors represented higher on a vertically oriented legend map to larger quantities. Here, we investigated cases in which these biases make opposing predictions, depending on the degree to which they operate at the level of *conceptual* magnitude represented in the colormap (e.g., healthiness), or the level of *numeric* magnitude used to measure that concept (e.g., rank order). We found conceptual magnitude consistently dominated for the high-is-more bias and tended to dominate for the dark-is-more bias unless the concept was less salient. Thus, efforts to create visualizations for effective communication cannot merely rely on software defaults for mapping numbers to colors; it is necessary to consider the meaning of the data.

## Introduction

Wh﻿en people communicate about data, they leverage perceptual representations to help make sense of patterns in datasets. Such perceptual representations include data *visualizations*, such as diagrams, charts, and maps (see Franconeri et al., 2021 for a review), data *sonifications* (audition) (Dingler et al., 2008; Mynatt, 1994), *tactilizations* (touch) (Jones, 2011), and even *olfactations* (smell) (Batch et al., 2020; Patnaik et al., 2018). In all of these cases, designers encode aspects of data (e.g., quantities or categories) using perceptual features (e.g., color, position, size, frequency, texture, or odor). Observers are then faced with the task of determining what those perceptual features mean in the context of the particular perceptual representation.

Colormap data visualizations are one common type of perceptual representation, which are used to display a wide variety of data types, such as weather patterns across different geographical regions, correlations in neural activity across different brain regions, and the spread of disease across the globe. In colormap data visualizations, variations of color are used to represent variations in magnitude within a dataset. When observers interpret colormaps, they have expectations about how colors should map to magnitude (Cuff, 1973; McGranaghan, 1989; Schloss et al., 2019; Schoenlein et al., 2023; Sibrel et al., 2020), known as their *inferred mappings.* Interpreting colormaps, and information visualizations more broadly, is easier when visualization design matches people’s inferred mappings (Hegarty, 2011; Lin et al., 2013; Mukherjee et al., 2022; Norman, 2013; Schloss et al., 2018, 2019, 2021; Schoenlein et al., 2023; Sibrel et al., 2020; Tversky, 2011). Thus, understanding the nature of people’s inferred mappings is fundamental to understanding how to design data representations that support effective communication.

Multiple factors influence inferred mappings for colormap data visualizations, including relational and direct associations. Relational associations are correspondences between relational properties of visual features (e.g., darkness, opacity, and spatial height) and relational properties of concepts (e.g., *more* or *less* of a concept). For example, the dark-is-more bias leads people to infer that darker colors map to larger quantities (Cuff, 1973; McGranaghan, 1989; Schloss et al., 2019; Schoenlein et al., 2023; Sibrel et al., 2020). And, the high-is-more bias leads people to infer that colors represented higher in a vertically oriented legend map to larger quantities (Schloss et al., 2019; Sibrel et al., 2020).^1^ This bias is consistent with the general notion that positions higher in a picture plane correspond to larger quantities (Hegarty, 2011; Tversky, 2011; Tversky et al., 1991). This phenomenon extends to gestures, as TV broadcasters tend to raise their hands vertically when they reference higher quantities (Winter et al., 2013). Other known relational associations for colormaps include the opaque-is-more bias (Schloss et al., 2019) and the hotspot-is-more bias (Schott, 2010; Sibrel et al., 2020). Direct associations are the degree to which a given concept is associated with a particular color (e.g., sunshine is strongly associated with light yellows but not with dark grays, whereas shade is strongly associated with dark grays but not light yellows). Direct associations can lead observers to infer that colors more associated with the concept (e.g., more sunshine, more shade) map to larger quantities, especially if those associations are particularly strong (Schoenlein, et al., 2023).

When multiple factors are activated, they combine to produce people’s inferred mappings for a particular visualization (Schloss et al., 2019; Schoenlein et al., 2023; Sibrel et al., 2020). Sometimes these factors work together, but sometimes they conflict and can cancel each other out. For example, when colormaps have a hotspot (concentric regions that form a sort of bull’s eye) and that hotspot is dark, the dark-is-more bias and hotspot-is-more bias work together, leading people to infer that darker regions map to more. But, when the hotspot is light, these two biases conflict. Depending on the salience of the hotspot, the dark-is-more bias can dominate, the two biases can cancel out, or the hotspot-is-more bias can dominate (Sibrel et al., 2020). Schoenlein et al. (2023) laid the groundwork for a method that can predict people’s inferred mappings from a weighted sum over multiple, sometimes competing factors.

**Fig. 1:**
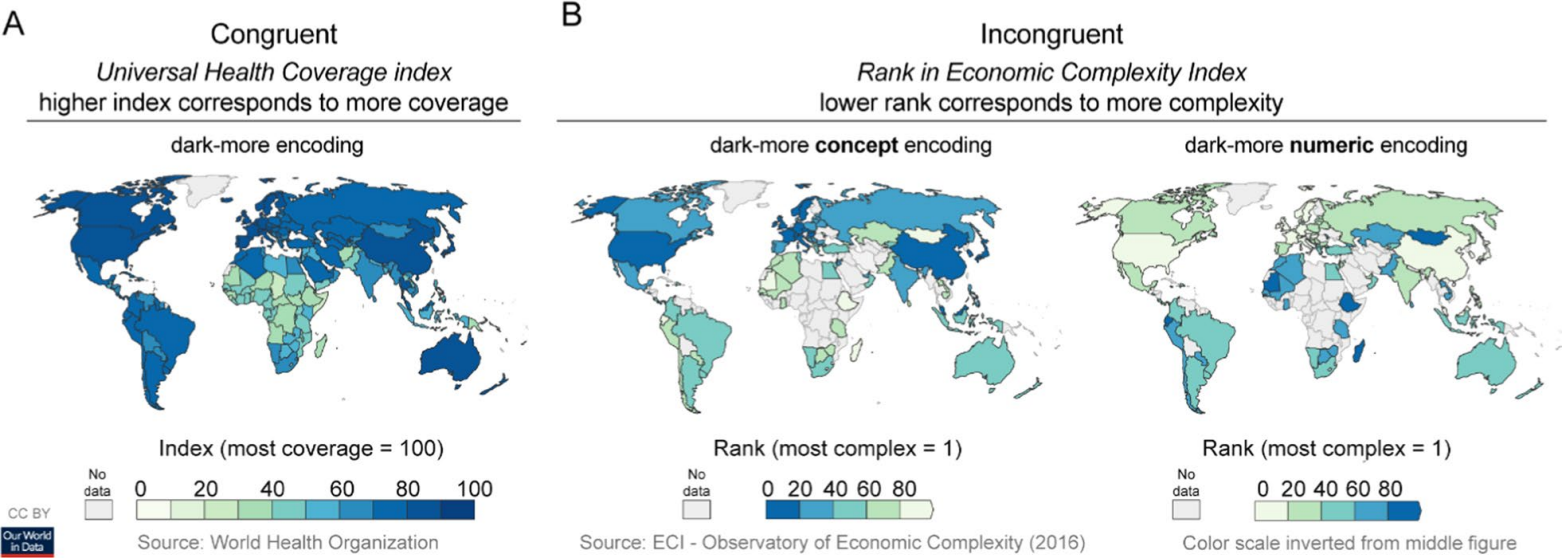
Example colormaps representing **A** congruent and **B** incongruent domain concepts. **A** Colormap of the Universal Health Coverage Index featuring a dark-more encoding (darker colors encode larger index values, which correspond to more coverage). Figure from Ortiz-Ospina and Roser (2017). **B** Two colormaps representing country ranks based on the Economic Complexity Index. In **B**, left (original figure), darker colors encode for more complexity (lower ranks, which are smaller numbers). In **B**, right, the color scale has been inverted, such that darker colors encode for less complexity (higher ranks, which are larger numbers). Figures adapted from Ortiz-Ospina and Beltekian (2018); Simoes and Hidalgo (2011)

However, when evaluating mappings between visual features and magnitude in colormap visualizations, there are two types of magnitude to consider, and previously it was unknown which type(s) of magnitude influence inferred mappings. The first type is *conceptual magnitude*, which is the amount of the construct represented in the visualization. The second type is *numeric magnitude,* which is the quantitative value measured when operationalizing the construct. This distinction is shown in Fig. 1, using data from the World Health Organization (Ortiz-Ospina & Beltekian, 2018; Ortiz-Ospina & Roser, 2017; Simoes & Hidalgo, 2011). In Fig. 1A, the construct is health coverage, which is operationalized with a health index. Here, conceptual and numeric magnitude are congruent because greater health coverage corresponds to larger health index values. In Fig. 1B (left), the construct is economic complexity, which is operationalized using a complexity ranking. Here, conceptual and numeric magnitudes are incongruent because more complexity corresponds to smaller numbers in the rank order.

In previous studies on inferred mappings for colormaps, conceptual and numeric magnitude were congruent (or the concept was too vague to determine). When they were congruent, it was implied that “more” of the concept corresponded to “more” of the numeric magnitude. For example, in Cuff (1973), colormaps represented temperature, such that increased temperature corresponded to larger degrees. In Schloss et al., (2019) and Sibrel et al., (2020), colormaps represented alien animal sightings on a fictitious planet, such that increased sightings corresponded to larger counts of animals (Schloss et al., 2019). In McGranaghan (1989), colormaps represented unspecified data, so congruency was too vague to determine.

Yet, as shown in Fig. 1B, cases arise in the real world in which conceptual and numeric magnitude conflict. In Fig. 1B (left), darker colors map to larger conceptual magnitude (greater economic complexity) and smaller numeric magnitude (rank position), whereas in Fig. 1B (right), darker colors map to smaller conceptual magnitude and larger numeric magnitude. Under such conflicts, are inferred mappings influenced by the conceptual level, the numeric level, or a combination of both levels? To address this question, we studied the dark-is-more bias and high-is-more bias under conditions when the conceptual and numeric level were congruent or incongruent.

## Study overview

**Fig. 2:**
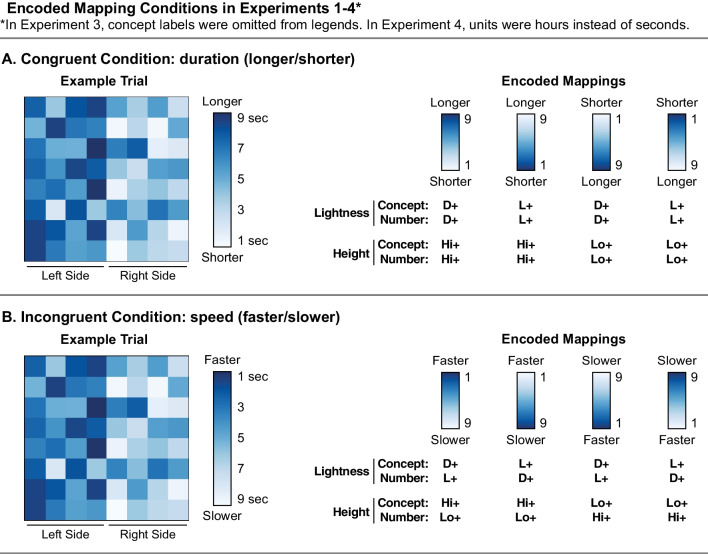
Encoded mapping conditions in Experiments 1–4 for the **A** congruent and **B** incongruent conditions. The example trials (left) correspond directly to Experiments 1 and 2. In Experiment 3 there were no concept labels on the legend, only numbers. In Experiment 4, the numeric units were hours rather than seconds. Within each condition, participants saw legends with four different encoded mappings, as shown in the legends on the right. With respect to the conceptual level, the legend conditions included 2 lightness encoded mappings (dark-more; D+ or light-more; L+) $\times$ 2 height encoded mappings (high-more; Hi+ or low-more; Lo+). Encoded mappings at the numeric level match the conceptual level in the congruent conditions and are opposite of the conceptual level in the incongruent conditions

All experiments in this study followed the experimental paradigm established in Schloss et al. (2019). Participants were presented with colormaps along with a legend (Fig. 2A, left). The legend specified the *encoded mapping* (i.e., the correspondence between visual features and magnitude in the colormap). The lightness encoded mapping varied such that larger magnitude in the data corresponded to darker colors (dark-more; D+) or lighter colors (light-more; L+). The height encoded mapping also varied such that colors that were higher on a vertically oriented legend map to larger quantities (high-more; Hi+) or colors that were lower on the legend mapped to larger quantities (Lo+). Participants were asked to look at the colormap and legend and to indicate which side of the map had more (or less) of a target concept. We assessed response times (RT) to correctly interpret the legend. It is established that observers are faster at interpreting colormaps when the encoded mapping more closely matches their inferred mappings, so we can learn about inferred mappings by determining which kinds of encoded mappings enable faster RTs (Schloss et al., 2019; Sibrel et al., 2020).

In previous studies using this paradigm, the legend was labeled to indicate which endpoint represented “greater” and which endpoint represented “fewer” (conceptual magnitude) but there were no numbers on the legend (numeric magnitude) (Schloss et al., 2019; Sibrel et al., 2020). Here, we included numbers on the legend so we could vary the congruency of the conceptual and numeric magnitude. We will first describe our general approach in terms of the conditions in Experiment 1, and then describe how Experiments 2–5 adapted this procedure.

In Experiment 1, participants were presented with colormaps depicting fictitious data about the amount of time alien animals took to notice a scientist observing them in different regions of a planet (adapted from Schloss et al., 2019; Sibrel et al., 2020). All participants were told that time was measured in terms of seconds, and all participants saw legends that were labeled from 1 sec. to 9 sec. Congruency varied between subjects. For the congruent group, the instructions described time in terms of *duration,* and the legends were labeled with 1 sec. as “shorter” and 9 sec. as “longer” (Fig. 2A). Thus, greater duration corresponded to larger numeric values. The target concept was “longer,” such that participants were asked to look at the map and decide whether the time it took the animals to notice they were being observed was *longer* on the left or right side of the observation site. The encoded mapping at the conceptual level always matched the encoded mapping at the numeric level (e.g., both D+ or both L+ for lightness encoded mapping and both Hi+ or both Lo+ for height encoded mapping).

For the incongruent group, the instructions described time in terms of *speed*, and the legends were labeled with 1 sec. as “faster” and 9 sec. as “slower.” Thus, more speed corresponded to smaller numeric values of time. The target concept was “faster,” such that participants were asked to look at the map and decide whether the time it took the animals to notice they were being observed was *faster* on the left or right side of the observation site. The encoded mapping at the conceptual level was always mismatched with the encoded mapping at the numeric level (e.g., one was D+ and the other was L+ for lightness encoded mapping and one was Hi+ and the other was Lo+ for height encoded mapping). We acknowledge that speed entails time relative to distance and we only indicate speed in terms of time, but we chose this condition because response time is often described in terms of speed (faster/slower) in psychological studies.

**Fig. 3:**
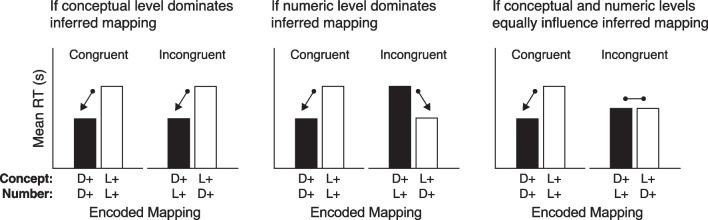
Potential patterns of results for the congruent and incongruent conditions depending on whether the conceptual level dominates inferred mappings (left), the numeric level dominates (middle) or the conceptual and numeric levels equally play a role (right). The y-axis represents mean response time (RT) in seconds. The labels along the x-axis correspond to lightness encoded mapping and indicate whether the encoded mapping was dark-more (D+) or light-more (L+) at the conceptual or numeric level (see Fig. 2 for examples of legends with these different encoded mappings). These predictions also apply to height encoded mapping if D+ were replaced with high-more (Hi+) and L+ were replaced with low-more (Lo+)

Figure 3 shows potential patterns of results depending on whether the dark-is-more bias operates at the conceptual level (left), numeric level (middle), or an equal combination of both (right). For the congruent condition (conceptual and numeric levels both have dark-more (D+) encoding or light-more (L+) encoding), both levels can work together.^2^ Thus, we expect RTs in the congruent condition will be faster for dark-more encoding than light-more encoding (extending Schloss et al., 2019; Sibrel et al., 2020). For the incongruent condition, the pattern of results will depend on the relative strength of the conceptual and numeric levels. If the conceptual level dominates inferred mappings, RTs will be faster when the *conceptual level* is encoded as dark-more, even though the numeric level is encoded as light-more. If the numeric level dominates, RTs will be faster when the *numeric level* is coded as dark-more, even though the conceptual level is encoded as light-more. If both levels play a role, then they may cancel out as shown in Fig. 3 (right), such that RTs will be similar for both conditions. Figure 3 is shown with respect to the dark-is-more bias, but the same patterns apply to the high-is-more bias if dark-more (D+) is replaced with high-more (Hi+) and light-more (L+) is replaced with low-more (Lo+).

Experiments 2–5 were variations of Experiment 1 to test the generalizability of the results (Table 1). In Experiment 2, the displays were the same as Experiment 1, but the instructions were different. Instead of being asked about the *more* endpoint of the conceptual dimension (“longer” for duration, “faster” for speed), participants were asked about the *less* endpoint (“shorter” for duration, “slower” for speed). In Experiment 3, the instructions were the same as Experiment 1, but the conceptual magnitude was made less salient by omitting conceptual magnitude labels from the legend and only showing numeric magnitude. In Experiment 4, the data domain was changed from alien animals to antibiotic discovery. Participants were told that the colormaps represented data about the amount of time it took microbes to eliminate pathogens from a Petri dish. This scenario is based on real research in the Tiny Earth Project, which aims to discover new antibiotics to address the decline in effective antibiotics (Hurley et al., 2021). The numeric magnitude unit was changed from seconds to hours, but otherwise the experiment displays were the same as Experiment 1. Finally, in Experiment 5, the data domain was changed to public health. Participants were told that the colormaps represented health data in different counties, similar to the County Health Rankings report, an annual report of the physical and mental well-being of communities throughout states in the US ("County Health Rankings & Roadmaps, 2022"). The target concept was healthiness for both the congruent and incongruent conditions, but in the congruent condition, healthiness was quantified as an index (larger numbers indicated healthier), and in the incongruent condition, healthiness was quantified as a rank order (smaller numbers indicated healthier).

**Table 1:** Overview of Experiments 1–5

Exp	Data domain	Legend	Congruency	Construct	Unit	Target concept	Target magnitude
1	Alien animals	Concepts, #	Congruent Incongruent	Duration Speed	Sec Sec	“Longer” “Faster”	More More
2	Alien animals	Concepts, #	Congruent Incongruent	Duration Speed	Sec Sec	“Shorter” “Slower”	Less Less
3	Alien animals	# only	Congruent Incongruent	Duration Speed	Sec Sec	“Longer” “Faster”	More More
4	Antibiotics	Concepts, #	Congruent Incongruent	Duration Speed	Hr Hr	“Longer” “Faster”	More More
5	Public health	Concepts, #	Congruent Incongruent	Healthiness Healthiness	Index Rank	“Healthier” “Healthier”	More More

While creating the stimuli, we tried to avoid factors that would influence participant responses, beyond those we aimed to test. We used fictitious data in abstract scenarios to avoid cases in which participants have domain knowledge that could influence their responses. We tested domains in which participants were unlikely to have strong direct associations that would override the dark-is-more bias (alien animals, antibiotics, public health). We created colormaps that do not have strong perceptual evidence for opacity variation (see Schloss et al., 2019) and they were presented on a light background, so we focused on the dark-is-more bias and high-is-more bias and did not consider the potential effects of the opaque-is-more bias. The colormaps also did not have spatial cues in the data, such as hotspots often found in weather and neuroimaging data, so we did not consider potential effects of the hotspot-is-more bias (Sibrel et al., 2020).

The stimuli, data, and analysis code for all experiments can be found at https://osf.io/kpqjh.

## Experiment 1

Experiment 1 assessed the degree to which inferred mappings operated at the conceptual or numeric level for colormaps representing sightings by alien animals. In the congruent condition, participants were told that the data represented duration, and participants judged whether the time was longer on the left/right side of the map. In the incongruent condition, participants were told that the data represented speed, and they judged whether the time was faster on the left/right side of the map.

### Methods

#### Participants

We aimed for 60 participants (30 per group), based on the sample sizes in Schloss et al. (2019) and Sibrel et al. (2020). We collected data in batches (*n* = 85 total) until reaching at least 30 participants per group after excluding participants for atypical color vision (*n* = 8) and for accuracy less than 90% (*n* = 16; exclusion criteria set following Schloss et al. (2019) to ensure there were sufficient accurate trials to assess effects on response time). Color vision was assessed by asking participants: “Do you have difficulty seeing colors or noticing differences between colors compared to the average person?” and “Do you consider yourself to be colorblind?” The final sample was 61 participants (32 women, 29 men, mean age = 18.46; age and gender reported through open-response text fields in all experiments). All participants in this and all subsequent experiments participated online for extra credit in their Introductory Psychology course at the University of Wisconsin–Madison. Each experiment tested a different set of participants, all of whom were from an Introductory Psychology course within a single academic year. All gave informed consent, and the University of Wisconsin-Madison IRB approved the protocol.

#### Design and displays

As shown in Fig. 2A and B (left), the display for each trial contained a colormap visualization and a legend (stimuli adapted from Schloss et al., 2019). The colormap visualization (referred to as “colormap” for short) was an 8 $\times$ 8 grid (4.8 cm $\times$ 4.8 cm) placed in the center of the screen. These dimensions pertain to a 7 in. $\times$ 11.25 in. monitor (2560 $\times$ 1600 resolution) but can vary depending on the monitor size of individual participants. Each cell in the grid represented fictitious data about the amount of time it took for alien animals to notice they were being observed by a scientist. To help participants categorize the data coming from the left and right sides of the colormap, the left four columns were labeled “left side” and the right four columns were labeled “right side.”

**Fig. 4:**
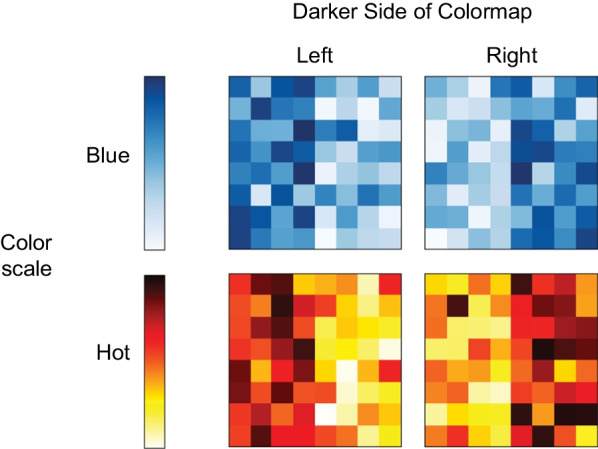
Two color scales, ColorBrewer Blue, “Blue” for short, and Hot from MATLAB, and example colormap stimuli constructed from the Blue and Hot color scales (left/right balancing which side is darker)

The legend included a color scale (3.5 cm tall $\times$ 0.5 cm wide) displayed 1 cm to the right of the colormap (also known as a color ramp as in Smart et al. (2019)). To the right of the color scale were numeric time labels: “1 sec.,” “3,” “5,” “7,” and “9 sec.” For the congruent group, the concept label “longer” (more of the concept) was next to “9 sec.” and “shorter” (less of the concept) was next to “1 sec.” (Fig. 2A). For the incongruent group, the concept label “faster” (more of the concept) was next to the numeric label “1 sec.,” and “slower” (less of the concept) was next to “9 sec.” (Fig. 2B). The colormap and legend were positioned on a white rectangle (13 cm $\times$ 8 cm) centered on a medium gray background (RGB = [128, 128, 128]). The colormaps were generated using two possible color scales (Fig. 4): Hot (from MATLAB) and ColorBrewer Blue (“Blue” for short) (Harrower & Brewer, 2003).

Each colormap was constructed using a different underlying dataset to help ensure that the results were not due to particular spatial patterns of squares within any one colormap. The datasets were created by sampling eight points along an arctangent curve with added noise sampled from a normal distribution (see Schloss et al., 2019 for details). This approach resulted in colormaps in which one side was biased to be light and the other side was biased to be dark. Within each color scale participants judged 40 colormaps (treated as repetitions), 20 colormaps with the darker side on the left and 20 with the darker side on the right. Thus, there were 80 unique colormap stimuli: 2 color scales (Hot, Blue) $\times$ 2 darker sides (left/right) $\times$ 20 underlying datasets.

Participants saw each of these 80 colormap conditions four times, corresponding to four different legend conditions: 2 lightness encodings at the conceptual level (dark-more, light-more) $\times$ 2 height encoded mappings at the conceptual level (high-more, low-more) (Fig. 2). In the congruent condition, the numeric encoded mapping matched the conceptual encoded mapping (Fig. 2A), and in the incongruent condition, the numeric encoded mapping was opposite of the conceptual encoded mapping (Fig. 2B). This design also ensured that the orientation of the color scale in the legend was balanced over trials, such that the darker end was higher on half of the trials and lower on the other half.

In total, each participant completed 320 experimental trials, including these 4 legend conditions [2 lightness encoded mappings (D+, L+) $\times$ 2 height encoded mappings (Hi+, Lo+)] presented with each of the 80 colormap stimuli. Congruency varied between subjects, with random assignment to the congruent group (*n* = 30) or the incongruent group (*n* = 31).

#### Procedure

All participants were instructed that they would see colormaps that represent data collected by a scientist on a distant planet, Sparl. They were told that the data were about alien animals from different regions of observation sites and how much time the animals took to notice the scientist. In the congruent group, participants were told that in some observation sites, the time it took was LONGER on the left side of the site, and in other sites, the time was LONGER on the right side. Their task was to look at the colormap and legend, and indicate if the time it took the animals to notice they were being observed was LONGER on the left or right side of the observation site. In the incongruent group, the word “LONGER” was replaced with the word “FASTER”, but otherwise the instructions were the same. At the bottom of the screen, four example colormaps were displayed so participants could see the types of stimuli they would be asked to judge. The full set of instructions can be found in the Additional file 1.

After reading the instructions, participants completed 20 practice trials, randomly sampled from all possible trials. They then began the 320 experiment trials. Each trial started with a 500-ms blank gray screen with a black fixation cross in the center. Next, the experimental display appeared and remained on the screen until the participant responded (pressing the left/ right arrow key). If they responded correctly, the next trial appeared after a 500-ms delay. If they responded incorrectly, black text that said “WRONG” appeared 2 cm to the right of the legend for 500 ms, followed by a blank 500-ms screen, and then the next trial began. Participants were notified when they completed 25%, 50%, and 75% of the trials, and were told their accuracy after every 20 trials. At the end of the experiment, participants were presented two color vision questions as described above, followed by a debriefing message to inform them of the purpose of the experiment.

### Results and discussion

To prepare the response time (RT) data for analysis, we excluded incorrect trials, and then pruned trials for each participant if RTs were + /–2 standard deviations from their mean over all trials. Next, for each participant, we calculated the mean RT over the remaining trials within each of the four legend conditions for each color scale (also averaging over the left/right balance of which side of the colormaps was darker).

We analyzed the full dataset using a mixed-design ANOVA: 2 congruency groups (congruent vs. incongruent, between-subjects) $\times$ 2 lightness encoded mappings (dark-more concept vs. light-more concept; within-subjects) $\times$ 2 height encoded mappings (high-more concept vs. low-more concept; within-subjects) $\times$ 2 color scales (hot vs. blue, within-subjects). Note: for analysis purposes we coded encoded mappings in reference to the concept, rather than the numeric magnitude, but this was an arbitrary decision. In the Additional file 1: Table S1 shows the full output of the analysis and Additional file 1: Figure S1 shows the data plotted according to lightness encoded mapping and height encoded mapping for each color scale.^3^ Here, we examine the effects of congruency on the dark-is-more bias and high-is-more bias separately because there was no 3-way interaction between congruency, lightness encoded mapping, and height encoded mapping, (*F*(1,59) = 1.58, *p* = 0.214, ${\eta }_{p}^{2}$ = 0.026).

#### Dark-is-more bias

**Fig. 5:**
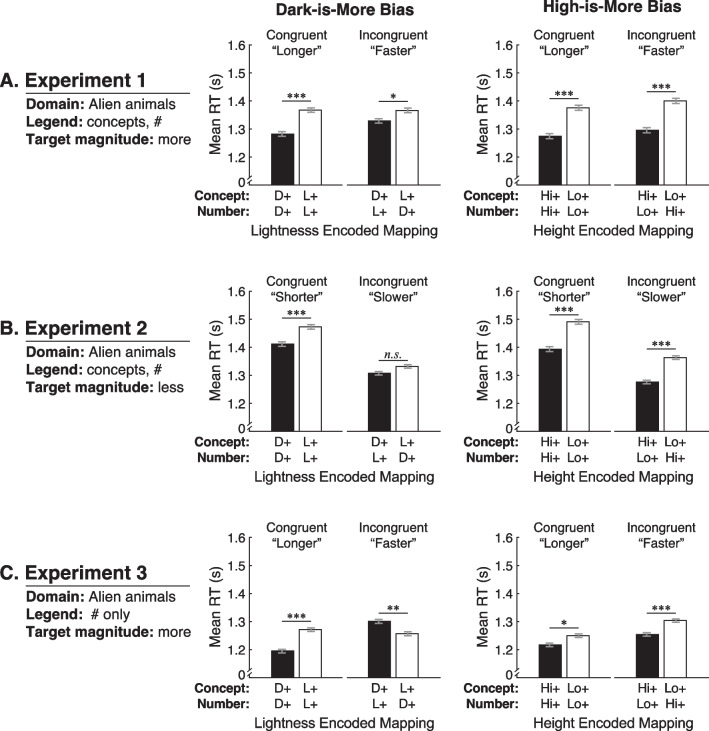
Mean response times (RTs) in **A** Experiment 1, **B** Experiment 2, and **C** Experiment 3. In the column labeled “Dark-is-More Bias,” mean RTs are shown separately for dark-more (D+, black bars) and light-more (L+, white bars) lightness encoded mappings at the conceptual and numeric levels, averaged over height encoded mapping and color scale. In the column labeled “High-is-More Bias,” mean RTs are shown for high-more (Hi+, black bars) and low-more (Lo+, white bars) height encoded mappings at the conceptual and numeric levels, averaged over lightness encoded mapping and color scale. Within each plot, the bars are labeled according to congruency (congruent/incongruent) along with the corresponding target concept for that group in each experiment (“longer”/ “faster” in Experiments 1 and 3,” “shorter”/ “slower” in Experiment 2. Error bars represent the standard error of the means (SEMs) calculated using the Cousineau (2005) adjustment to account for subject-level differences in RT

Figure 5A (left) shows mean RTs plotted for trials with dark-more concept encoding and light-more concept encoding, separated by whether participants were in the congruent or incongruent group. These data are averaged over height encoded mapping and color scale. The pattern of results is similar to the pattern if the conceptual level dominated inferred mappings (Fig. 3, left).

Consistent with a dark-is-more bias operating at the conceptual level, a main effect of lightness encoded mapping indicated quicker RTs for dark-more concept encoding than light-more concept encoding (*F*(1,59) = 29.45, *p* < 0.001, ${\eta }_{p}^{2}$ = 0.333). However, lightness encoded mapping interacted with congruency (*F*(1,59) = 4.43, *p* = 0.040, ${\eta }_{p}^{2}$ = 0.070). As shown in Fig. 5A (left), the degree to which RTs were quicker for dark-more than light-more concept encoding was greater for the congruent group than the incongruent group. Given this interaction, we conducted paired-samples *t* tests to compare the effects of encoded mapping separately within each group. RTs were quicker for dark-more concept encoding in both the congruent group (*t*(29) = − 5.22, *p* < 0.001, *d*_z_ = 0.953) and incongruent group (*t*(30) = − 2.40, *p* = 0.023, *d*_z_ = 0.430). Taken together, these results suggest that the dark-is-more bias operates primarily at the conceptual level, but the numeric level does play a role. Conflict from the numeric level reduces the effect of the conceptual level, but the dark-is-more bias at the conceptual level still dominated inferred mappings.

#### High-is-more bias

Figure 5A (right) shows mean RTs plotted for trials with high-more concept encoding and low-more concept encoding, separated by whether participants were in the congruent group or incongruent group. These data are averaged over lightness encoding and color scale. Consistent with a high-is-more bias operating at the conceptual level, a main effect of height encoded mapping indicated quicker RTs for high-more concept encoding than low-more concept encoding (*F*(1,59) = 65.06, *p* < 0.001, ${\eta }_{p}^{2}$= 0.524). This effect did not interact with congruency (*F* < 1), which suggests there was no interference from when the numerical level conflicted (i.e., larger conceptual magnitude and lower numeric magnitude were positioned higher on the legend). Paired-samples *t* tests indicated that RTs were quicker for high-more encoding than low-more encoding for both the congruent group (*t*(29) = − 5.64, *p* < 0.001, *d*_z_ = 1.029) and the incongruent group (*t*(30) = − 5.78, *p* < 0.001, *d*_z_ = 1.037).

In summary, the results of Experiment 1 suggest that both the dark-is-more and high-is-more biases are dominated by the conceptual level. Under conflicts between conceptual and numeric levels, the numeric level plays a role for the dark-is-more bias, but not enough to eliminate or override the bias at the conceptual level. No such interference occurred for the high-is-more bias. These results suggest that when assigning perceptual features to quantities in colormap data visualizations, it is important to consider what the data mean, and not just numeric values, to facilitate interpretation.

## Experiment 2

Experiment 2 assessed whether the results of Experiment 1 were robust to the framing of the task. Instead of asking participants about the “more” endpoint of the conceptual dimension— “longer” in the duration (congruent) group, “faster” in the speed (incongruent) group, we modified the instructions to ask about the “less” endpoint of the conceptual dimension— “shorter” in the duration (congruent) group, “slower” in the speed (incongruent) group. Otherwise, Experiment 2 was the same as Experiment 1.

### Methods

#### Participants

We collected data from 92 new participants to reach a target sample of 60 participants, after excluding participants for atypical color vision (*n* = 7) and < 90% accuracy (*n* = 25). The final sample included 40 women and 20 men (mean age = 18.82), who were randomly assigned to the congruent group (*n* = 30) or the incongruent group (*n* = 30).

#### Design, displays, and procedure

The methods were the same as Experiment 1, except for a modification to the instructions. In the congruent group, the word “LONGER” was replaced with the word “SHORTER” (e.g., was the time it took the animals to notice they were being observed by a scientist SHORTER on the left or right side of the observation site?). In the incongruent group the word “FASTER” was replaced with the word “SLOWER” (e.g., was the time it took the animals to notice they were being observed by a scientist SLOWER on the left or right side of the observation site?). The full instructions can be found in the Additional file 1.

### Results and discussion

We prepared the data for analysis and used the same mixed-design ANOVA as in Experiment 1. We report on the main results below and in Fig. 5B, plotted in the same manner as in Experiment 1. In the Additional file 1: Table S1 shows the full output of the analysis and Figure S2 shows the data plotted according to lightness encoded mapping and height encoded mapping for each color scale. None of the effects beyond those reported below were statistically significant.

#### Dark-is-more bias

Similar to Experiment 1, a main effect of lightness encoding indicated that mean RTs were quicker for dark-more concept encoding than light-more concept encoding (*F*(1,58) = 17.02, *p* < 0.001, ${\eta }_{p}^{2}$ = 0.227) (Fig. 5B, left). Unlike Experiment 1, there was no significant interaction between encoded mapping and congruency (*F*(1,58) = 3.13, *p* = 0.082, ${\eta }_{p}^{2}$ = 0.051), suggesting the dark-is-more bias operated at the conceptual level with no interference from conflicting numeric magnitude. Paired-samples *t* tests indicated that RTs were quicker for dark-more encoding than light-more encoding in the congruent group (*t*(29) = − 3.73, *p* < 0.001, *d*_z_ = 0.681) but this difference did not reach statistical significance in the incongruent group (*t*(29) = − 1.92, *p* = 0.065, *d*_z_ = 0.351). Taken together, this set of analyses suggests that the dark-is-more bias was primarily driven by the conceptual level. Although the effect seemed weaker in the incongruent condition, it was not different enough from the congruent condition to cause an interaction.

#### High-is-more bias

As in Experiment 1, a main effect of height encoded mapping indicated that mean RTs were quicker for high-more conceptual encoded than low-more conceptual encoding (*F*(1,58) = 73.21, *p* < 0.001, ${\eta }_{p}^{2}$ = 0.558) (Fig. 5B, right). This effect did not significantly interact with congruency (*F* < 1) suggesting no interference from the numeric level. Paired-samples *t* tests indicated that RTs were quicker for high-more encoding than low-more encoding for both the congruent group (*t*(29) = − 5.61, *p* < 0.001, *d*_z_ = 1.024) and the incongruent group (*t*(29) = − 6.82, *p* < 0.001, *d*_z_ = 1.245).

Examining the RTs overall in Fig. 5A and B, it appeared that RTs might be slower in Experiment 2 than in Experiment 1. However, an independent-samples *t* test showed no significant difference in RTs between the two experiments (*t*(119) = − 0.68, *p* = 0.497, *d* = 0.124).

In summary, Experiment 2 suggested that the dark-is-more bias and high-is-more bias operated at the conceptual level with no interference from the numeric level. These results were similar to Experiment 1, even though the task probed the “less” endpoint of the conceptual dimension. This similarity suggests that these biases reflect a true correspondence between perceptual and conceptual dimensions, and are not an artifact of probing the greater or lesser endpoint of the conceptual dimension in the experimental task.

## Experiment 3

In Experiment 3, we assessed whether the dark-is-more bias and high-is-more bias would still operate at the conceptual level if the conceptual level was less salient during the task. To address this question, we removed the concept labels from the endpoints of the legends. Otherwise, this experiment was the same as Experiment 1.

### Methods

#### Participants

We collected data from 83 new participants before reaching a target sample of 60 participants after excluding participants for atypical color vision (*n* = 7) and < 90% accuracy (*n* = 16). The final sample included 36 women and 24 men (mean age = 18.70), who were randomly assigned to either the congruent group (*n* = 30) or the incongruent group (*n* = 30).

#### Design, displays, and procedure

The methods were the same as Experiment 1, except we removed the concept labels at the endpoints of the legend so that only the numbers remained. Thus, the congruent and incongruent groups saw the same experimental displays. The conceptual magnitude was only mentioned in the instructions, when the congruent group was asked to judge if the time it took animals to notice they were being observed by a scientist was LONGER on the left or right side of the observation site, and the incongruent group was asked to judge if the time it took animals to notice they were being observed by a scientist was FASTER on the left or right side of the observation site.

### Results and discussion

We prepared the data for analysis and used the same mixed-design ANOVA as in Experiment 1. We report on the main results below and in Fig. 5C, plotted in the same manner as in Experiment 1. In the Additional file 1: Table S1 shows the full output of the analysis and Figure S3 shows the data plotted according to lightness encoded mapping and height encoded mapping for each color scale. None of the effects beyond those reported below were statistically significant.

#### Dark-is-more bias

Figure 5C (left) shows mean RTs plotted in the same manner as Fig. 5A–B, but the data look quite different. The pattern of results is similar to the pattern if the numeric level dominated inferred mappings (Fig. 3, middle).

Unlike Experiments 1 and 2, there was no main effect of lightness encoded mapping (*F*(1,58) = 2.92, *p* = 0.093, ${\eta }_{p}^{2}$ = 0.048) but lightness encoded mapping strongly interacted with congruency (*F*(1,58) = 41.50, *p* < 0.001, ${\eta }_{p}^{2}$ = 0.417). To understand this interaction, we conducted paired-samples *t* tests within each group. In the congruent group, participants responded quicker when there was dark-more concept encoding, which also corresponds to dark-more *numeric* encoding (*t*(29) = − 6.02, *p* < 0.001, *d*_*z*_ = 1.100). In the incongruent group, participants responded quicker when there was light-more concept encoding, corresponding to dark-more *numeric* encoding (*t*(29) = 3.21,* p* = 0.003, *d*_*z*_ = 0.587). Taken together, these results suggest that when no legend text was present to remind participants of the concept represented in the colormaps, the dark-is-more bias operated at the numeric level rather than the conceptual level.

#### High-is-more bias

As in Experiments 1 and 2, RTs were quicker when the implied location of the label indicating more was higher on the legend, even though that label was not actually present on the legend (*F*(1,58) = 21.85, *p* < 0.001, ${\eta }_{p}^{2}$ = 0.274) (Fig. 5C, right). Legend text position did not interact with congruency (*F* < 1). Paired-samples *t* tests indicated that RTs were quicker for high-more encoding than low-more encoding for both the congruent group (*t*(29) = − 2.55, *p* = 0.016, *d*_z_ = 0.466) and the incongruent group (*t*(29) = − 4.11, *p* < 0.001, *d*_z_ = 0.751). Thus, the high-is-more bias operated at the conceptual level, even when the conceptual level was less salient due to removing the concept labels from the legend.

Examining the overall RTs in Fig. 5A and C, it appeared that RTs might be quicker in Experiment 3 than Experiment 1. However, an independent-samples *t* test showed no significant difference in RTs between the two experiments (*t*(119) = 1.43, *p* = 0.155, *d* = 0.260).

In summary, the results of Experiment 3 suggest that when the conceptual level is less salient, the dark-is-more bias operates at the numeric level rather than the conceptual level, but the high-is-more bias still operates at the conceptual level. These results suggest that the high-is-more bias may be more deeply ingrained in the conceptual level than is the dark-is-more bias. We return to this possibility in the General Discussion.

## Experiment 4

In Experiments 1–3, we studied inferred mappings using a fictitious scenario about alien animals. In Experiment 4, we attempted to replicate the results of Experiment 1 in a more ecologically valid scenario: antibiotic discovery. Participants were told the colormaps represented data about the time it took microbes collected by a scientist to eliminate pathogens from a Petri dish. This scenario is based on real-world research in a project called Tiny Earth led by Dr. Jo Handelsman at the University of Wisconsin-Madison. The Tiny Earth project aims to “studentsource” antibiotic discovery to address the diminishing supply of effective antibiotics (Hurley et al., 2021).

### Methods

#### Participants

We collected data from 82 new participants to reach a target sample of 60 participants after excluding participants for atypical color vision (*n* = 8) and < 90% accuracy (*n* = 14). The final sample included 29 women and 31 men (mean age = 18.62 years), who were randomly assigned to either the congruent group (*n* = 30) or the incongruent group (*n* = 30).

#### Design, displays, and procedure

The methods were the same as Experiment 1, except for the scenario presented to participants in the instructions. The participants were told that a scientist gathered soil samples from different farms to examine in her laboratory. Different farms have different microbes that could become antibiotics. This is an important task because bacteria are starting to evolve into “superbugs,” which are resistant to our most-used antibiotics. In the congruent group, participants were asked if the time it took the microbes to eliminate pathogens from the Petri dish was LONGER on the left or right side of the given farm represented in each colormap. In the incongruent group, the word LONGER was replaced with FASTER. The full sets of instructions can be found in the Additional file 1. As in Experiments 1 and 2, we included the concept labels and numeric values on the legend.

### Results and discussion

**Fig. 6:**
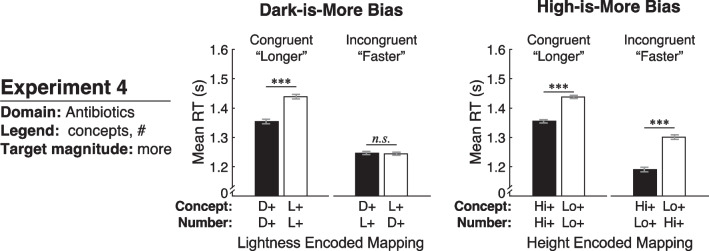
Results of Experiment 4, plotted in the same manner as in Fig. 5 for Experiments 1–3

We prepared the data for analysis and used the same mixed-design ANOVA as in Experiment 1. We report on the main results below and in Fig. 6, plotted in the same manner as in Experiment 1. In the Additional file 1, Table S2 shows the full output of the analysis and Figure S4 shows the data plotted according to lightness encoded mapping and height encoded mapping for each color scale.^4^

#### Dark-is-more bias

As shown in Fig. 6 (left), mean RTs in this experiment resemble the pattern of results if both the conceptual level and numeric level influence inferred mappings (Fig. 4, right). A main effect of lightness encoded mapping indicated that RTs were quicker for dark-more encoding at the conceptual level (*F*(1,58) = 18.90, *p* < 0.001, ${\eta }_{p}^{2}$ = 0.246), but this effect interacted with congruency (*F*(1,58) = 21.15, *p* < 0.001, ${\eta }_{p}^{2}$= 0.267). Paired-samples *t* tests indicated that RTs were significantly quicker for dark-more than light-more encoding in the congruent group (*t*(29) = − 5.37, *p* < 0.001, *d*_*z*_ = 0.981), but this difference was not significant in the incongruent group (*t*(29) = 0.23, *p* = 0.882, *d*_z_ = 0.041). Thus, when there was a conflict between the dark-is-more bias at the conceptual and numeric levels, neither the conceptual level nor the numeric level dominated inferred mappings.

This lack of effect in the incongruent condition could be related to the especially quick RTs in that group. That is, there was a main effect of congruency (*F*(1,58) = 4.15, *p* = 0.046, ${\eta }_{p}^{2}$ = 0.067), with quicker RTs to report where microbes were *faster* at eliminating bacteria (incongruent group) than when they were asked where microbes took *longer* to eliminate bacteria. Responding according to *faster* may have been more intuitive and therefore easier for participants because it was better aligned with the domain goal of antibiotic discovery to eliminate pathogens. These quick RTs may have dampened any effects of the dark-is-more bias.

#### High-is-more bias

As in Experiments 1–3, RTs were quicker for high-more encoding than low-more encoding (*F*(1,58) = 100.11, *p* < 0.001, ${\eta }_{p}^{2}$= 0.633), see Fig. 6 (right). Again, height encoded mapping did not significantly interact with congruency (*F*(1,58) = 2.06, *p* = 0.157, ${\eta }_{p}^{2}$ = 0.034), suggesting that the high-is-more bias operated at the conceptual level for the congruent and incongruent groups. Paired-samples *t* tests indicated that RTs were quicker for high-more encoding than low-more encoding for both the congruent group (*t*(29) = − 7.37, *p* < 0.001, *d*_z_ = 1.346) and the incongruent group (*t*(29) = − 7.03, *p* < 0.001, *d*_z_ = 1.284). Here, the faster overall RTs for the incongruent group did not seem to dampen the effects of the high-is-more bias, as observed for the dark-is-more bias. This difference could be due to the high-is-more bias being generally stronger than the dark-is-more bias.

Overall, there was no significant difference in RTs comparing Experiment 4 with Experiment 1 (*t*(119) = 0.268, *p* = 0.789, *d* = 0.049).

In summary, the results of this experiment replicated some, but not all of the findings from Experiment 1 in a more ecologically valid scenario: antibiotic discovery. Overall, there was a dark-is-more bias at the level of the concept, but this effect was driven by the congruent condition with no effect of lightness encoding in the incongruent condition. The lack of effect in the incongruent condition suggests that the dark-is-more bias at the numeric level could have been strong enough to cancel out the dark-is-more bias at the conceptual level. It is also possible that the extremely quick RTs in the incongruent condition dampened any effects of the dark-is-more bias. The results for the high-is-more bias were more straightforward. As in all the previous experiments, the high-is-more bias operated at the conceptual level, with no interference from the numeric level. Given inconsistencies in the present results for the dark-is-more bias across experiments, we sought to extend our findings to yet another domain, public health.

## Experiment 5

In Experiment 5, we attempted to replicate the results of Experiment 1 in another ecologically valid scenario: public health across different counties. In the congruent group, participants were told the data represented a health index, in which larger numbers indicated more health (e.g., the healthiest county had an index of 101). In the incongruent group, participants were told that the data represented a health ranking, in which lower ranks indicated more health (e.g., the healthiest county had a rank of 1).

### Methods

#### Participants

We collected data from 76 new participants to reach a target sample of 60 participants after excluding participants for atypical color vision (*n* = 5) and < 90% accuracy (*n* = 11). The final sample included 42 women and 18 men (mean age = 18.5 years), who were randomly assigned to either the congruent group (*n* = 30) or the incongruent group (*n* = 30).

#### Design, displays, and procedure

**Fig. 7:**
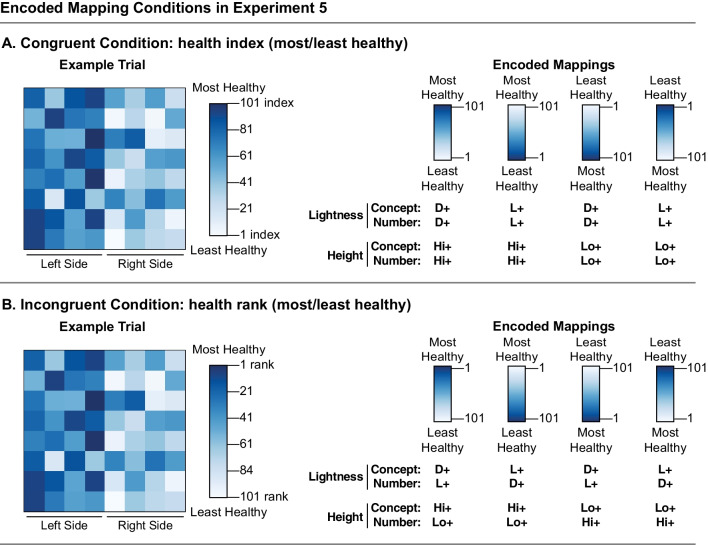
Encoded mapping conditions in Experiment 5 for the **A** congruent and **B** incongruent conditions. Participants in both conditions indicated whether populations were healthier on the left or right side of the map. In the congruent condition, healthiness was quantified using an index, such that larger numbers corresponded to greater health. In the incongruent condition, healthiness was quantified using a rank ordering, such that smaller numbers corresponded to greater health. The encoded mappings were analogous to Experiments 1–4 (Fig. 2)

The design and displays were similar to Experiment 1, with the following exceptions. Participants were told that the colormaps represented fictitious data from a health INDEX (congruent group) or a health RANK (incongruent group). The legend numbers and labels were changed to fit this scenario about health data. The number scale next to the legend displayed the numbers “1,” “21,” “41,” “61,” “81,” and “101” (Fig. 7). In this experiment, both groups saw the same concept labels on top of the legends: “most healthy” and “least healthy.” For the congruent group, the label “most healthy” was next to the number 101, and “least healthy” was next to the number 1. For the incongruent group, these pairings were switched, so that “most healthy” was next to the number 1 and “least healthy” was next to the number 101. Metric type (rank/index) was included next to “1” and “101” on the numeric scale, rather than time units (sec./hr) as in the previous experiments.

Participants were instructed that they would see colormaps representing data collected by a public health researcher about populations of people in different counties across the state. The populations varied in a health index/health ranking. Participants were asked to indicate whether populations were healthier on the left or right side of the colormap. The full set of instructions can be found in the Additional file 1.

### Results and discussions

**Fig. 8:**
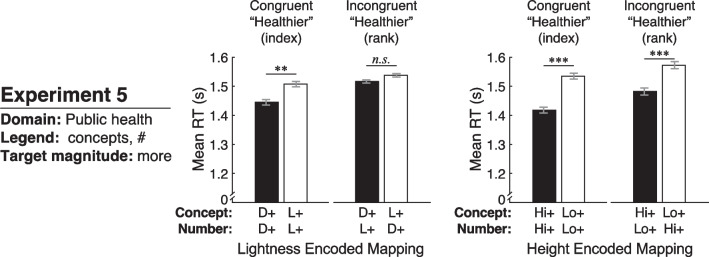
Results of Experiment 5, plotted in the same manner as in Fig. 5 for Experiments 1–3 and Fig. 6 for Experiment 4

We prepared the data for analysis and used the same mixed-design ANOVA as in Experiment 1. We report on the main results below and in Fig. 8, plotted in the same manner as in Experiment 1. In the Additional file 1: Table S2 shows the full output of the analysis and Figure S5 shows the data plotted according to lightness encoded mapping and height encoded mapping for each color scale.^5^

#### Dark-is-more bias

As shown in Fig. 8 (left), mean RTs in this experiment look like a blend between the pattern of results if the conceptual level dominated inferred mappings (Fig. 3, left) and both the conceptual level and numeric level equally influenced inferred mappings (Fig. 3, right). A main effect of lightness encoded mapping indicated that RTs were quicker for dark-more concept encoding than light-more concept encoding (*F*(1,58) = 15.39, *p* < 0.001, ${\eta }_{p}^{2}$ = 0.210). This effect did not significantly interact with congruency (*F*(1,58) = 3.84, *p* = 0.055, ${\eta }_{p}^{2}$ = 0.062). Paired-samples *t* tests indicated that RTs were quicker for dark-more encoding than light-more encoding in the congruent group (*t*(29) = − 3.43, *p* = 0.002, *d*_z_ = 0.626) but this difference did not reach statistical significance in the incongruent group (*t*(29) = − 1.91, *p* = 0.066, *d*_z_ = 0.349). Taken together, this set of analyses suggest that the dark-is-more bias was primarily driven by the conceptual level. Although the effect seemed weaker in the incongruent condition, it was not different enough from the congruent condition to cause an interaction.

#### High-is-more bias

As in the previous four experiments, the high-is-more bias was dominated by the conceptual level (Fig. 8, right). A main effect of height encoded mapping indicated that RTs were quicker for high-more concept encoding than low-more concept encoding (*F*(1,58) = 44.93, *p* < 0.001, ${\eta }_{p}^{2}$ = 0.437). This factor did not interact with congruency (*F* < 1), suggesting no interference from the numeric level. Paired-samples *t* tests indicated that RTs were quicker for high-more encoding than low-more encoding for both the congruent group (*t*(29) = − 5.98, *p* < 0.001, *d*_z_ = 1.091) and the incongruent group (*t*(29) = − 3.77, *p* < 0.001, *d*_z_ = 0.689). Thus, the high-is-more bias operated primarily at the conceptual level for both groups.

Examining the RTs overall in Figs. 5A and 7, it appeared that RTs might be slower in Experiment 5 than in Experiment 1. This observation was supported by an independent-samples *t* test comparing mean RTs for Experiment 1 versus Experiment 5 (*t*(119) = − 2.32, *p* = 0.022, *d* = 0.422). This increased response time for Experiment 5 may be due to the increased complexity of the legends in Experiment 5, which included two-word concept labels (most healthy, least healthy), and condition labels next to the numeric scale (rank, index) rather than unit labels (sec., hour).

In summary, the dark-is-more bias was dominated by the conceptual level. Although some evidence suggested that the conflicting numeric level may have weakened this effect in the incongruent condition, the difference was not strong enough to cause an interaction between lightness encoded mapping and congruency. As in all of the preceding experiments, the high-is-more bias operated at the conceptual level, with no interference from the numeric level.

## General discussion

The goal of this study was to investigate the degree to which inferred mappings between visual features and concepts operate at the conceptual and/or numeric level. We addressed this question by studying two biases known to influence inferred mappings for colormap data visualizations, the dark-is-more bias and high-is-more bias. We examined a variety of data types and domains: response times of alien animals to notice scientists (Experiments 1–3), response times of microbes to eliminate pathogens for antibiotic discovery (Experiment 4), and public health metrics across various counties (Experiment 5).

When conceptual and numeric magnitude were congruent, our results replicated previous work demonstrating the dark-is-more bias (quicker RTs for dark-more encoding) and the high-is-more bias (quicker RTs for high-more encoding) (Schloss et al., 2019; Sibrel et al., 2020). These patterns persisted when the target concept in the experimental task was the larger endpoint of the conceptual dimension (Experiments 1, 3–5), or the smaller endpoint (Experiment 2). This finding suggests that these biases reflect a true correspondence between perceptual and conceptual dimensions and are not merely due to probing either the greater or lesser endpoint of the conceptual dimension in the experimental task.

When conceptual and numeric magnitude were incongruent, we could assess the distinct effects of the conceptual and numeric levels on inferred mappings. Across all five experiments, the high-is-more bias operated at the conceptual level, with no significant interference from the numeric level. That is, it was generally easier to interpret colormaps when larger conceptual magnitude mapped to colors positioned higher on the vertically oriented legend, even when larger numeric values were lower on the legend.

However, for the dark-is-more bias, the results for the incongruent condition varied across experiments. First, we consider the four experiments in which the conceptual level was labeled in the legend (Experiments 1–2 and 4–5). In three out of the four experiments in which the conceptual level was labeled (Experiments 1, 2, and 5, but not Experiment 4) the value of mean RTs for the incongruent condition was quicker for dark-more encoding at the conceptual level than at the numeric level, but that difference only reached statistical significance in Experiment 1. Experiment 4 was also unique in that RTs were especially quick in the incongruent condition which may have dampened any effects of the dark-is-more bias. This observation was supported by a main effect of congruency in Experiment 4, which was not found in the other experiments. In considering why the incongruent (*faster*) condition had especially quick RTs in Experiment 4, one possibility is that there is a value judgment on the data represented in the colormaps that influenced RTs. In the scenario described in the instructions of Experiment 4, the desired outcome is for microbes to eliminate pathogens faster (rather than take longer), so this framing of the task may have facilitated participant responses. In the scenario described in the instructions of Experiments 1–2, it is not clear whether the desired outcome is for alien animals to notice they are being observed faster, or to take longer, so the framing may have had less of an effect. In Experiment 5, the target concept was the same (“healthier”) for both the congruent and incongruent groups, so there is no difference in value judgment. These observations suggest future studies are needed to understand how value judgments about data represented in visualizations may influence the effects of inferred mappings on interpretations of visualizations.

The only case in which the dark-is-more bias significantly dominated at the *numeric level* was in Experiment 3, when the conceptual magnitudes were not labeled on the legends and were only mentioned in the instructions. This result suggests that people need to be reminded of the conceptual level for it to have an effect. However, we caution readers against concluding that in the absence of concept labels, dark-more encoding at the numeric level will be easiest to interpret in real-world scenarios. In our study, the instructions describing the conceptual level were only provided at the beginning of the experiment, and then participants completed hundreds of trials without a reminder of the concepts represented in the visualization. In real-world scenarios, when colormaps are presented on the news, in research presentations, or in written reports, they are often accompanied by verbal descriptions that remind observers about concepts represented in the visualization. These verbal descriptions may activate the conceptual level, even when labels are absent. Further work is needed to understand how such additional information might influence inferred mappings.

In this study, we investigated two of many factors known to influence inferred mappings and found that the high-is-more bias consistently operated at the level of conceptual magnitude and the dark-is-more bias was more variable. A question that stems from this finding is, why does this difference arise? One possibility is that high-is-more is a stronger bias, and therefore less susceptible to variation, but why might the high-is-more bias be stronger?

Although the origins of these biases are still unknown, some have argued that they may stem from ecological statistics. The high-is-more bias may stem from observations that height increases as objects in the world accumulate or grow, like accumulating paperwork on a desk or growing children as they age (Tversky, 2011). The high-is-more bias may also be reinforced by frequent exposure to its effects in daily life, such as observing TV broadcasters gesture higher up when talking about larger quantities (Winter et al., 2013). The dark-is-more bias may stem from observations that regions tend to get darker with greater accumulation, like ink on a page or birds in a flock (Cuff, 1973; McGranaghan, 1989). These observations may be strong tendencies in the world, but there are exceptions. For example, deeper (more) ocean water goes *lower* underground, and accumulating white snow on a dark pavement makes the ground appear *lighter*.

Evidence against the high-is-more bias in the real-world could be less abundant or less visible (i.e., underground) than evidence against the dark-is-more bias, making the high-is-more bias more robust. In fact, differences in the apparent lightness of accumulating objects (depending on whether objects are dark/light and the background is dark/light) have been purported to result in an entirely different bias—the opaque-is-more bias. In the opaque-is-more bias, observers infer that regions appearing more opaque (given the background color) map to larger quantities (Schloss et al., 2019). Critically, the opaque-is-more bias is not merely due to lightness contrast with the background because dark backgrounds do not lead observers to infer that lighter-colored regions map to larger quantities if the regions do not appear to vary in opacity (McGranaghan, 1989; Schloss et al., 2019). Future work is needed to better understand the origins of these biases, and whether their robustness can be explained by ecological regularities in the world.

Another open question concerning the robustness of the high-is-more bias is whether graph literacy might moderate the effect. The participants of the present experiments, all undergraduate college students, are assumed to have experience reading graphs, and so may have a level of graph literacy that prompts expectations about how graph legends are structured. Future work could investigate the high-is-more bias with people who have less familiarity with graphs, including children, to determine whether graph literacy plays a role.

## Conclusions

In conclusion, this study suggests that understanding people’s inferences about the meaning of visual features in data visualizations, and perhaps perceptual features in data representations across modalities more broadly, requires considering the concepts represented by the data, and not just the numeric values. Across all five experiments, the high-is-more bias consistently operated at the level of conceptual magnitude, not numeric magnitude. There was also a tendency for the dark-is-more bias to operate at the level of conceptual magnitude, but numeric magnitude sometimes interfered, and could override conceptual magnitude when conceptual magnitude was less salient in the visualization. These results contribute to the understanding of people’s inferences about the meanings of colors in information visualizations, while expanding knowledge on factors relevant to design information visualizations that facilitate visual communication.

## Supplementary Information

**Additional file 1.** Additional file 1 includes tables reporting the full ANOVA output for Experiments 1-3 and Experiments 4-5, figures showing mean response time separated by color scale for Experiments 1-5, and participant instructions in each condition for Experiments 1-5.

## Data Availability

The stimuli, datasets, and analysis code supporting the conclusions for all five experiments of this article are publicly available at Open Science Framework (OSF): https://osf.io/kpqjh.

## Footnotes

1 We use the term “high-is-more bias” in reference to a phenomenon identified in prior work on colormaps but not referred to by name (Schloss et al., 2019; Sibrel et al., 2020).

2 When the conceptual and numeric levels are congruent, the same pattern of responses would arise regardless of whether the conceptual level dominates, the numeric level dominates, or both play a role.

3 None of the additional effects reported in the Additional file 1 were significant except for a 3-way interaction between lightness encoded mapping, height encoded mapping, and color scale (*F*(1,59) = 9.61, *p* = .003, ${\eta }_{p}^{2}$ = .140). As seen in Additional file 1: Figure S1, this interaction appears to be due to a larger effect of lightness encoded mapping for the blue color scale than the hot color scale within the high-more height encoded mapping condition, but less so within the low-more encoded mapping condition.

4 The only significant effect beyond those reported here was an interaction between lightness encoded mapping and height encoded mapping, where the dark-is-more bias was larger for high-more height concept encoding than low-more concept encoding (*F*(1,58) = 9.80, *p* = .003, ${\eta }_{p}^{2}$ = .145). Analogous interactions were previously reported in Schloss et al. (2019) and Sibrel et al. (2020).

5 The only significant effect beyond those reported here was an interaction between lightness encoded mapping and height encoded mapping, in which the dark-is-more bias was larger for high-more height concept encoding than low-more concept encoding, as in Experiment 4 (*F*(1,58) = 4.73, *p* = .034, ${\eta }_{p}^{2}$ = .075).
