# Supplementary material for: More of what? Dissociating effects of conceptual and numeric mappings on interpreting colormap data visualizations
Source: Cogn Res Princ Implic. 2023 Jun 19;8:38. doi: 10.1186/s41235-023-00482-1 (PMC10279625; doi:10.1186/s41235-023-00482-1)
Supplement: Supplementary file 1 — Additional file 1. Additional file 1 includes tables reporting the full ANOVA output for Experiments 1-3 and Experiments 4-5, figures showing mean response time separated by color scale for Experiments 1-5, and participant instructions in each condition for Experiments 1-5. [file 41235_2023_482_MOESM1_ESM.pdf]

Additional file 1

More of what? Dissociating effects of conceptual and numeric mappings on interpreting colormap data visualizations

Alexis Soto[1,3], Melissa A. Schoenlein[2,3], & Karen B. Schloss[2,3]
[1]Department of Integrative Biology, University of Wisconsin-Madison
[2]Department of Psychology, University of Wisconsin-Madison
[3]Wisconsin Institute for Discovery, University of Wisconsin-Madison

---

Supplementary Tables

Table S1. Output for full ANOVA models for Experiments 1-3 (2 congruency conditions [congruent vs. incongruent] $\times$ 2 lightness encoded mappings [dark-more concept vs. light-more concept] $\times$ 2 height encoded mappings [high-more concept vs. low-more concept] $\times$ 2 color scales [Blue vs. Hot]). Degrees of freedom for Experiment 1 were (1,59) and for Experiments 2 and 3 were (1,58).

	**Experiment 1**			**Experiment 2**			**Experiment 3**		
	F	*p*	*η2*	*F*	*p*	*η2*	*F*	*p*	*η2*
ColorScale	.390	.535	.007	.185	.669	.003	2.615	.111	.043
Congruency	.062	.804	.001	1.789	.186	.030	.465	.498	.008
Light	29.447	***	.333	17.022	***	.227	2.917	.093	.048
Height	65.064	***	.524	73.211	***	.558	21.855	***	.274
*Colorscale*Congruency*	1.479	.229	.024	.012	.912	.000	3.309	.074	.054
*Height*Congruency*	.021	.884	.000	.240	.626	.004	.894	.348	.015
*Light*Congruency*	4.426	.040*	.070	3.131	.082	.051	41.498	***	.417
*Colorscale *Height*	.038	.845	.001	.103	.749	.002	.404	.528	.007
*Colorscale*Light*	.645	.425	.011	.740	.393	.013	.908	.345	.015
*Height*Light*	.479	.491	.008	.663	.419	.011	.494	.485	.008
*Colorscale*Height *Congruency*	.196	.659	.003	.756	.388	.013	.998	.322	.017
*Colorscale*Light *Congruency*	.122	.728	.002	1.817	.183	.030	.012	.914	.000
*Height*Light *Congruency*	1.577	.214	.026	.419	.520	.007	3.490	.067	.057
*ColorScale*Height *Light*	9.611	.003**	.140	.086	.770	.001	.025	.875	.000
*Colorscale*Height *Light*Congruency*	1.329	.254	.022	1.651	.204	.028	.149	.701	.003

Table S2. Output for full ANOVA models as in Table S1 for Experiments 4-5 (2 congruency conditions [congruent vs. incongruent] × 2 lightness encoded mappings [dark-more concept vs. light-more concept] × 2 height encoded mappings [high-more concept vs. low-more concept] × 2 color scales [Blue vs. Hot]). Degrees of freedom for both experiments were all (1,58).

	**Experiment 4**			**Experiment 5**		
	F	*p*	*η2*	*F*	*p*	*η2*
ColorScale	1.241	.270	.021	3.115	.083	.051
Congruency	4.146	.046*	.067	.213	.646	.004
Light	18.902	***	.246	15.390	***	.210
Height	100.114	***	.633	44.932	***	.437
*Colorscale*Congruency*	1.618	.208	.027	.073	.788	.001
*Height*Congruency*	2.059	.157	.034	.745	.391	.013
*Light*Congruency*	21.153	***	.267	3.841	.055	.062
*Colorscale*Height*	.277	.601	.005	.006	.937	.000
*Colorscale*Light*	.046	.831	.001	1.223	.273	.021
*Height*Light*	9.798	.003**	.145	4.731	.034*	.075
*Colorscale*Height *Congruency*	1.879	.176	.031	.897	.348	.015
*Colorscale*Light *Congruency*	2.453	.123	.041	.006	.941	.000
*Height*Light *Congruency*	.010	.920	.000	.016	.900	.000
*ColorScale*Height *Light*	.006	.938	.000	.048	.827	.001
*Colorscale*Height *Light*Congruency*	.015	.903	.000	.001	.979	.000

Supplementary Figures

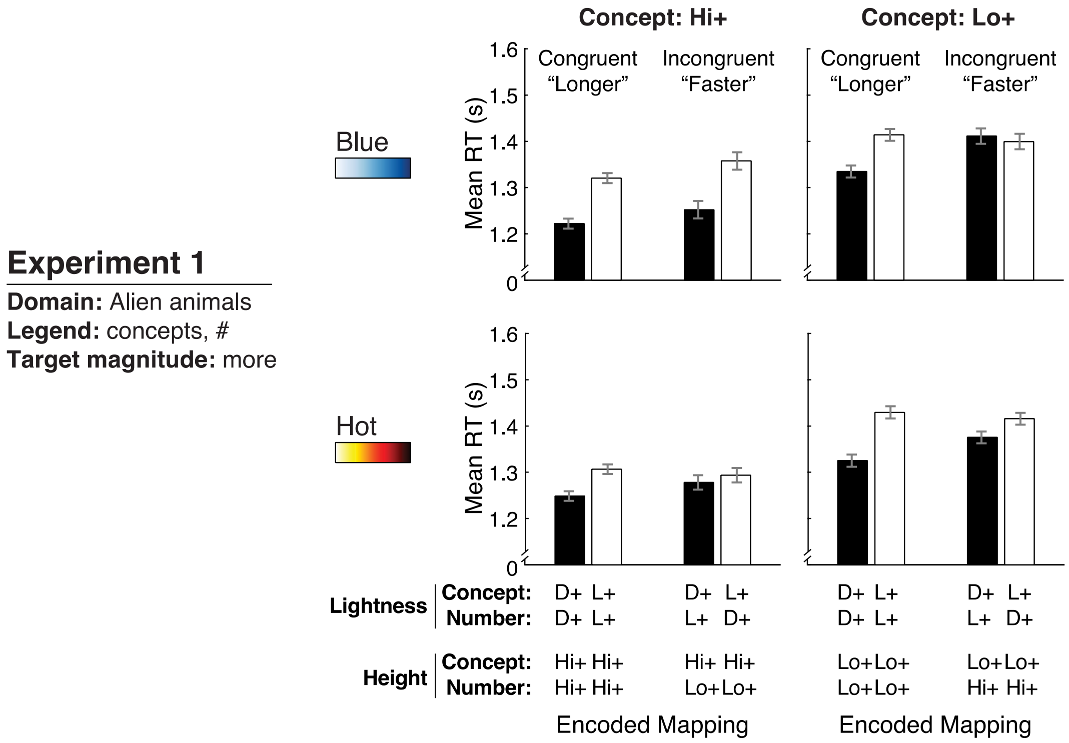

Figure S1. Mean response times (RTs) from Experiment 1 for the congruent (left pair of bars in each plot) and incongruent (right pair of bars in each plot) groups, for trials in which the colormaps were generated from the Blue color scale (top row) and Hot color scale (bottom row). RTs are further separated by trials in which colors higher on the legend mapped to more of the concept (Hi+, left column) or less of the concept on the legend (Lo+, right column), and when the legend specified a dark-more encoding (D+, black bars) or a light-more encoding (L+, white bars). Error bars represent standard error of the means (SEMs) calculated using the Cousineau (2005) adjustment to account for subject-level differences in RT.

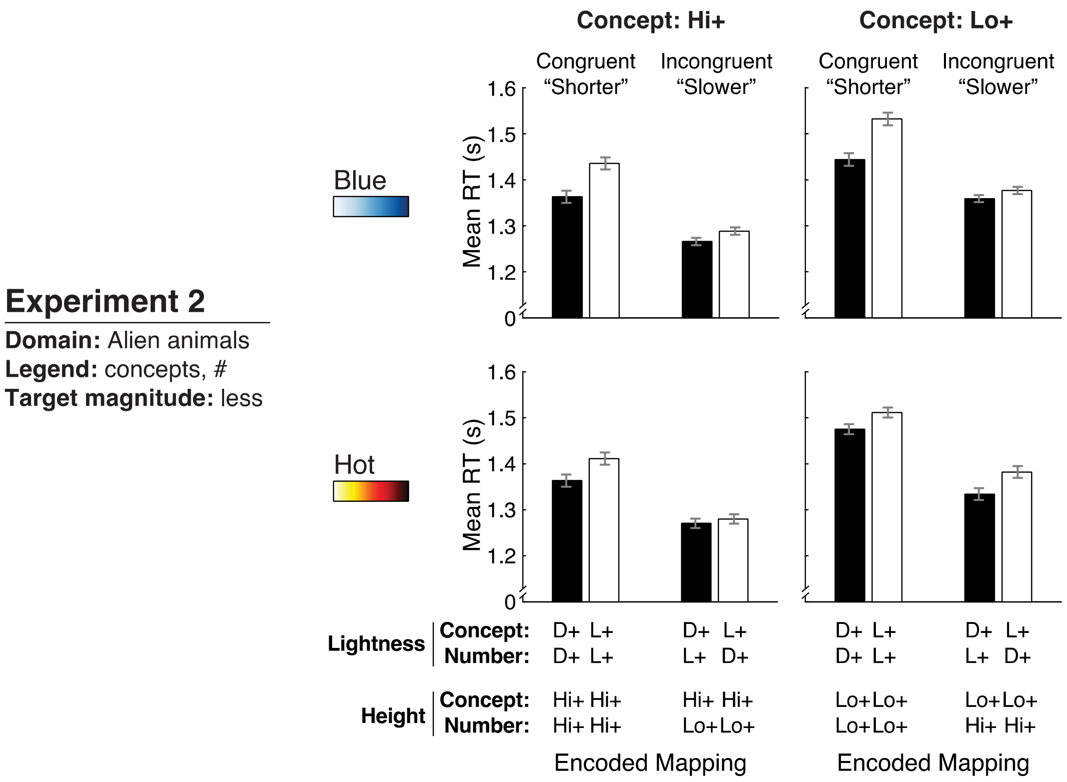

Figure S2. Mean response times from Experiment 2 plotted in the same manner as Figure S1.

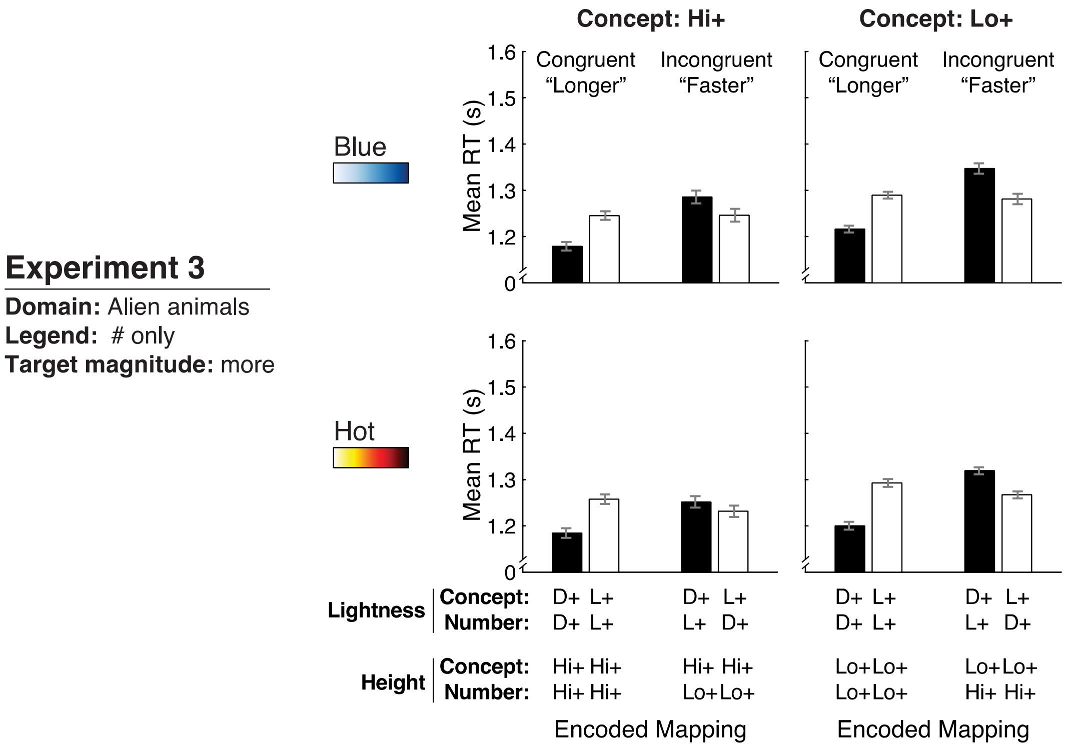

Figure S3. Mean response times from Experiment 3 plotted in the same manner as Figure S1.

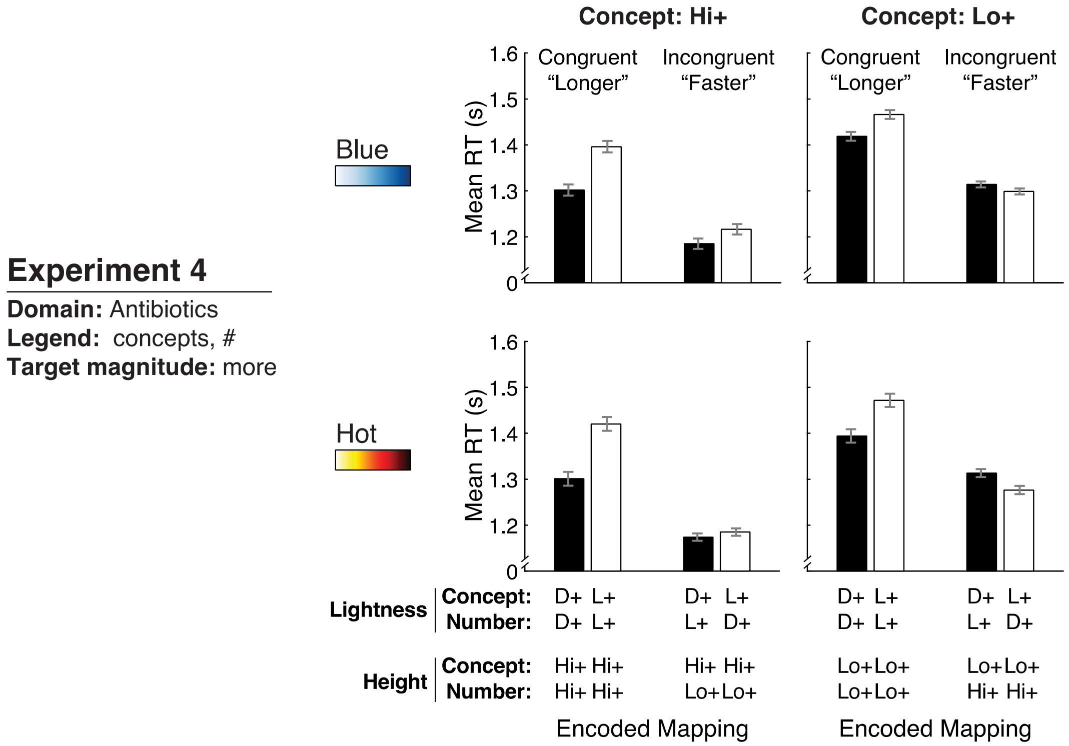

Figure S4. Mean response times from Experiment 4 plotted in the same manner as Figure S1.

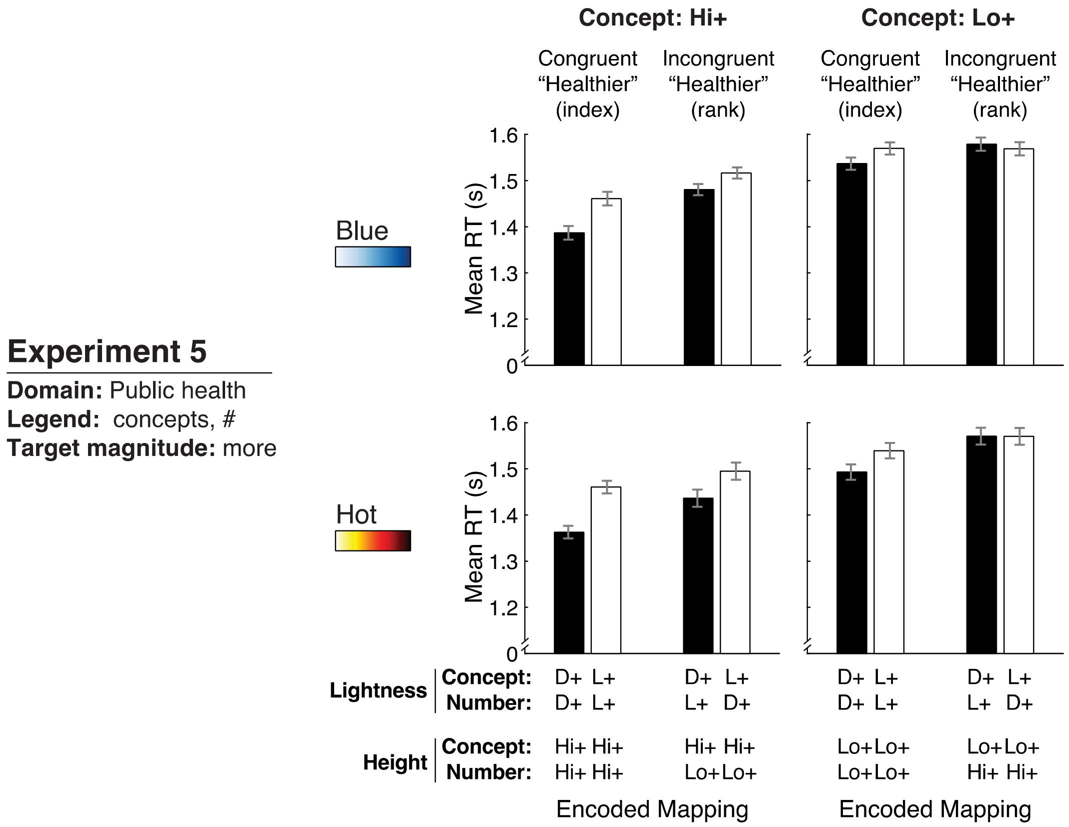

Figure S5. Mean response times from Experiment 5 plotted in the same manner as Figure S1.

Participant instructions for Experiments 1-5

Experiment 1 and Experiment 3

Congruent condition
In this experiment, you will see colormaps like the ones below. These colormaps show data collected by a scientist on a distant planet, Sparl. The scientist observed the behavior of alien animals at different observation sites across the planet.

Animals in different regions of each observation site varied in how much time they took to notice the scientist. In some cases, the time it took animals to notice the scientist in regions on the left side of the observation site was LONGER, and in other cases the time it took animals to notice the scientist in regions on the right side of the observation site was LONGER.

Each colormap displays the scientist's report of how much time it took for the animals of a given observation site to notice they were being observed. Each map has a legend that represents time in seconds. Aspects of the legend will vary trial to trial, so be sure to read the legend on every trial. Each trial will contain one colormap.

Your task is to look at the colormap and decide whether the time it took the animals to notice they were being observed was LONGER on the left or right side of the observation site. If the time the animals took to notice the scientist was LONGER on the left side, press the left arrow key. If the time the animals took to notice the scientist was LONGER on the right side, press the right arrow key.

Please respond as quickly as possible while maintaining your accuracy. You will be notified of your percent accuracy periodically. There will first be 20 practice trials. Once you begin the experiment you will be notified when you have completed 25%, 50%, and 75% of the trials.

Incongruent condition
In this experiment, you will see colormaps like the ones below. These colormaps show data collected by a scientist on a distant planet, Sparl. The scientist observed the behavior of alien animals at different observation sites across the planet.

Animals in different regions of each observation site varied in how much time they took to notice the scientist. In some cases, the time it took animals to notice the scientist in regions on the left side of the observation site was FASTER, and in other cases the time it took animals to notice the scientist in regions on the right side of the observation site was FASTER.

Each colormap displays the scientist's report of how much time it took for the animals of a given observation site to notice they were being observed. Each map has a legend that represents time in seconds. Aspects of the legend will vary trial to trial, so be sure to read the legend on every trial. Each trial will contain one colormap.

Your task is to look at the colormap and decide whether the time it took the animals to notice they were being observed was
FASTER on the left or right side of the observation site. If the time the animals took to notice the scientist was FASTER on the left side, press the left arrow key. If the time the animals took to notice the scientist was FASTER on the right side, press the right arrow key.

Please respond as quickly as possible while maintaining your accuracy. You will be notified of your percent accuracy periodically. There will first be 20 practice trials. Once you begin the experiment you will be notified when you have completed 25%, 50%, and 75% of the trials.

Experiment 2

Congruent condition
In this experiment, you will see colormaps like the ones below. These colormaps show data collected by a scientist on a distant planet, Sparl. The scientist observed the behavior of alien animals at different observation sites across the planet.

Animals in different regions of each observation site varied in how much time they took to notice the scientist. In some cases, the time it took animals to notice the scientist in regions on the left side of the observation site was SHORTER, and in other cases the time it took animals to notice the scientist in regions on the right side of the observation site was SHORTER.

Each colormap displays the scientist's report of how much time it took for the animals of a given observation site to notice they were being observed. Each map has a legend that represents time in seconds. Aspects of the legend will vary trial to trial, so be sure to read the legend on every trial. Each trial will contain one colormap.

Your task is to look at the colormap and decide whether the time it took the animals to notice they were being observed was SHORTER on the left or right side of the observation site. If the time the animals took to notice the scientist was SHORTER on the left side, press the left arrow key. If the time the animals took to notice the scientist was SHORTER on the right side, press the right arrow key.

Please respond as quickly as possible while maintaining your accuracy. You will be notified of your percent accuracy periodically. There will first be 20 practice trials. Once you begin the experiment you will be notified when you have completed 25%, 50%, and 75% of the trials.

Incongruent condition
In this experiment, you will see colormaps like the ones below. These colormaps show data collected by a scientist on a distant planet, Sparl. The scientist observed the behavior of alien animals at different observation sites across the planet.

Animals in different regions of each observation site varied in how much time they took to notice the scientist. In some cases, the time it took animals to notice the scientist in regions on the left side of the observation site was SLOWER, and in other cases the time it took animals to notice the scientist in regions on the right side of the observation site was SLOWER.

Each colormap displays the scientist's report of how much time it took for the animals of a given observation site to notice they were being observed. Each map has a legend that represents time in seconds. Aspects of the legend will vary trial to trial, so be sure to read the legend on every trial. Each trial will contain one colormap.

Your task is to look at the colormap and decide whether the time it took the animals to notice they were being observed was SLOWER on the left or right side of the observation site. If the time the animals took to notice the scientist was SLOWER on the left side, press the left arrow key. If the time the animals took to notice the scientist was SLOWER on the right side, press the right arrow key.

Please respond as quickly as possible while maintaining your accuracy. You will be notified of your percent accuracy periodically. There will first be 20 practice trials. Once you begin the experiment you will be notified when you have completed 25%, 50%, and 75% of the trials.

Experiment 4

Congruent condition

In this experiment, you will see colormaps like the ones below. These colormaps show data collected by a scientist who gathered soil samples to examine in their lab. The scientist observed the properties of soil from different farms. Each farm was in a different county of the state.

Soil contains microbes that produce antibiotics. This is important given that several human pathogens have evolved resistance to our most-used antibiotics, the so-called "superbugs." Thus, the scientist tried to discover new antibiotic-producing microbes in soil in order to address the diminishing supply of effective antibiotics.

Soil from different farms contained different antibiotic-producing microbes. Soil from different regions of each farm contained microbes that varied in how much time it took to kill pathogens in a Petri dish. In some cases, the time it took the microbes to eliminate pathogens was LONGER when the soil came from the left side of the farm, and in other cases the time it took the microbes to eliminate pathogens was LONGER when the soil came from the right side of the farm.

Each colormap displays the scientist's report of how much time it took for the microbes from the soil of a given farm to eliminate pathogens. Each map has a legend that represents time in hours. Aspects of the legend will vary trial to trial, so be sure to read the legend on every trial. Each trial will contain one colormap.

Your task is to look at the colormap and decide whether the time it took the antibiotics to eliminate pathogens was LONGER when the soil came from the left or right side of the farm. If the time it took the pathogens to be eliminated was LONGER when the soil was from the left side of the farm, press the left arrow key. If the time it took the pathogens to be eliminated was LONGER when the soil was from the right side of the farm, press the right arrow key.

Please respond as quickly as possible while maintaining your accuracy. You will be notified of your percent accuracy periodically. There will first be 20 practice trials. Once you begin the experiment you will be notified when you have completed 25%, 50%, and 75% of the trials.

Incongruent condition

In this experiment, you will see colormaps like the ones below. These colormaps show data collected by a scientist who gathered soil samples to examine in their lab. The scientist observed the properties of soil from different farms. Each farm was in a different county of the state.

Soil contains microbes that produce antibiotics. This is important given that several human pathogens have evolved resistance to our most-used antibiotics, the so-called "superbugs." Thus, the scientist tried to discover new antibiotic-producing microbes in soil in order to address the diminishing supply of effective antibiotics.

Soil from different farms contained different antibiotic-producing microbes. Soil from different regions of each farm contained microbes that varied in how much time it took to kill pathogens in a Petri dish. In some cases, the time it took the microbes to eliminate pathogens was FASTER when the soil came from the left side of the farm, and in other cases the time it took the microbes to eliminate pathogens was FASTER when the soil came from the right side of the farm.

Each colormap displays the scientist's report of how much time it took for the microbes from the soil of a given farm to eliminate pathogens. Each map has a legend that represents time in hours. Aspects of the legend will vary trial to trial, so be sure to read the legend on every trial. Each trial will contain one colormap.

Your task is to look at the colormap and decide whether the time it took the antibiotics to eliminate pathogens was FASTER when the soil came from the left or right side of the farm. If the time it took the pathogens to be eliminated was FASTER when the soil was from the left side of the farm, press the left arrow key. If the time it took the pathogens to be eliminated was FASTER when the soil was from the right side of the farm, press the right arrow key.

Please respond as quickly as possible while maintaining your accuracy. You will be notified of your percent accuracy periodically. There will first be 20 practice trials. Once you begin the experiment you will be notified when you have completed 25%, 50%, and 75% of the trials.

Experiment 5

Congruent condition
In this experiment, you will see colormaps like the ones below. These colormaps show data collected by a public health researcher in a state of the country. The researcher collected data from populations of people in different counties across the state. The populations in different regions of each county varied in a health index measure.

The index ranged from 1 (least health) to 101 (most health). In some cases, the populations in regions on the left side of the county were healthier, and in other cases the populations in regions on the right side of the county were healthier.

Each colormap displays the health index for a subset of different populations of a given county. Each map has a legend that represents this health index. Aspects of the legend will vary trial to trial, so be sure to read the legend on every trial. Each trial will contain one colormap.

Your task is to look at the colormap and decide whether populations were healthier on the left or right side of the county. If the populations were healthier on the left side, press the left arrow key. If the populations were healthier on the right side, press the right arrow key.

Please respond as quickly as possible while maintaining your accuracy. You will be notified of your percent accuracy periodically. There will first be 20 practice trials. Once you begin the experiment you will be notified when you have completed 25%, 50%, and 75% of the trials.

Incongruent condition
In this experiment, you will see colormaps like the ones below. These colormaps show data collected by a public health researcher in a state of the country. The researcher collected data from populations of people in different counties across the state. The populations in different regions of each county varied in a health ranking measure.

The ranking ranged from 1 (most health) to 101 (least health). In some cases, the populations in regions on the left side of the county were healthier, and in other cases the populations in regions on the right side of the county were healthier.

Each colormap displays the health ranking for a subset of different populations of a given county. Each map has a legend that represents this health ranking. Aspects of the legend will vary trial to trial, so be sure to read the legend on every trial. Each trial will contain one colormap.

Your task is to look at the colormap and decide whether populations were healthier on the left or right side of the county. If the populations were healthier on the left side, press the left arrow key. If the populations were healthier on the right side, press the right arrow key.

Please respond as quickly as possible while maintaining your accuracy. You will be notified of your percent accuracy periodically. There will first be 20 practice trials. Once you begin the experiment you will be notified when you have completed 25%, 50%, and 75% of the trials.
